# Serological Evidence of Lyssaviruses among Bats on Southwestern Indian Ocean Islands

**DOI:** 10.1371/journal.pone.0160553

**Published:** 2016-08-08

**Authors:** Julien Mélade, Stewart McCulloch, Beza Ramasindrazana, Erwan Lagadec, Magali Turpin, Hervé Pascalis, Steven M. Goodman, Wanda Markotter, Koussay Dellagi

**Affiliations:** 1 Centre de Recherche et de Veille sur les Maladies Emergentes dans l’Océan Indien (CRVOI), Plateforme de Recherche CYROI, Sainte Clotilde, La Réunion, France; 2 Université de La Réunion, UMR PIMIT «Processus Infectieux en Milieu Insulaire Tropical», INSERM U1187, CNRS 9192, IRD 249. Plateforme de Recherche CYROI, Saint Denis, La Réunion, France; 3 Institut de Recherche pour le Développement (IRD), Sainte-Clotilde, La Réunion, France; 4 Center for Viral Zoonoses, Department of Medical Virology, School of Medicine, Faculty of Health Sciences, University of Pretoria, Pretoria, South Africa; 5 Association Vahatra, Antananarivo, Madagascar; 6 Institut Pasteur de Madagascar, Antananarivo, Madagascar; 7 Field Museum of Natural History, Chicago, Illinois, United States of America; CEA, FRANCE

## Abstract

We provide serological evidence of lyssavirus circulation among bats on southwestern Indian Ocean (SWIO) islands. A total of 572 bats belonging to 22 species were collected on Anjouan, Mayotte, La Réunion, Mauritius, Mahé and Madagascar and screened by the Rapid Fluorescent Focus Inhibition Test for the presence of neutralising antibodies against the two main rabies related lyssaviruses circulating on the African continent: *Duvenhage lyssavirus* (DUVV) and *Lagos bat lyssavirus* (LBV), representing phylogroups I and II, respectively. A total of 97 and 42 sera were able to neutralise DUVV and LBV, respectively. No serum neutralised both DUVV and LBV but most DUVV-seropositive bats (n = 32/220) also neutralised *European bat lyssavirus 1* (EBLV-1) but not *Rabies lyssavirus* (RABV), the prototypic lyssavirus of phylogroup I. These results highlight that lyssaviruses belonging to phylogroups I and II circulate in regional bat populations and that the putative phylogroup I lyssavirus is antigenically closer to DUVV and EBLV-1 than to RABV. Variation between bat species, roost sites and bioclimatic regions were observed. All brain samples tested by RT-PCR specific for lyssavirus RNA were negative.

## Introduction

Lyssaviruses (order: *Mononegavirales*, family: *Rhabdoviridae*) are RNA viruses with single-stranded, negative-sense genomes approximately 12 kb in length [1]. Fifteen distinct viral species, classified into three phylogroups, have been identified to date within the *Lyssavirus* genus [2, 3]. These are the prototypic *Rabies lyssavirus* (RABV) and the 14 genetically related species referred to as rabies-related viruses that cluster into different phylogroups; *Duvenhage lyssavirus* (DUVV), *European bat lyssavirus* type 1 (EBLV-1) and type 2 (EBLV-2), *Australian bat lyssavirus* (ABLV), *Irkut lyssavirus* (IRKV), *Aravan lyssavirus* (ARAV), *Khujand lyssavirus* (KHUV) and *Bokeloh bat lyssavirus* (BBLV) constitute phylogroup I; *Lagos bat lyssavirus* (LBV), *Mokola lyssavirus* (MOKV) and *Shimoni bat lyssavirus* (SHIV) form phylogroup II; *Ikoma lyssavirus* (IKOV) and *West Caucasian bat lyssavirus* (WCBV) make up phylogroup III, whilst the newly described *Lleida bat lyssavirus* (LLEBV) potentially constituting phylogroup IV [3, 4]. With the exceptions of MOKV and IKOV, all lyssaviruses have been isolated from bats [5, 6].

RABV, the type species of the genus, is the principal causative agent of human rabies, a fatal acute encephalitis claiming an estimated 59,000 lives annually, primarily affecting Africa and Asia [7]. The natural cyclic maintenance of RABV worldwide is principally via dogs and wild canids [8], while bats are seen as a reservoir only in the New World, where the virus is known to infect insectivorous and hematophagous species [9, 10]. Nowadays, the majority of cases of human exposure to rabies in the New World is related to contact with rabid bats [10, 11]. In contrast, among the 14 rabies-related viruses, only EBLV-1 and 2, ABLV, DUVV, IRKV and MOKV have caused sporadic human deaths [12].

Terrestrial rabies is endemic on Madagascar where no native canids occur and dogs are both maintenance reservoirs and vectors. The other islands of the SWIO are deemed terrestrial rabies free and no case of RABV infection being recorded in non-volant mammals [13]. With respect to rabies related viruses, only one study conducted between 2005 and 2010 on Madagascar, recorded antibodies to LBV and EBLV-1, being positive in frugivorous bats (*Eidolon dupreanum* and *Pteropus rufus*) and negative in the six tested insectivorous bat species (n = 92) [14]. No data has been reported on rabies related lyssavirus infections in bats from other SWIO islands [13].

We report the results of a serosurvey conducted on bats from SWIO islands based on the screening of neutralising antibodies against two rabies related viruses, namely LBV and DUVV, known to circulate among African bat populations [15, 16]. Our data show evidence that at least two lyssaviruses circulate among bats in the insular ecosystems of the SWIO region. This study is the first comprehensive analysis of bat exposure to lyssaviruses in this region and aims to strengthen available information.

## Materials and Methods

### Specimen collection

**Fig 1:**
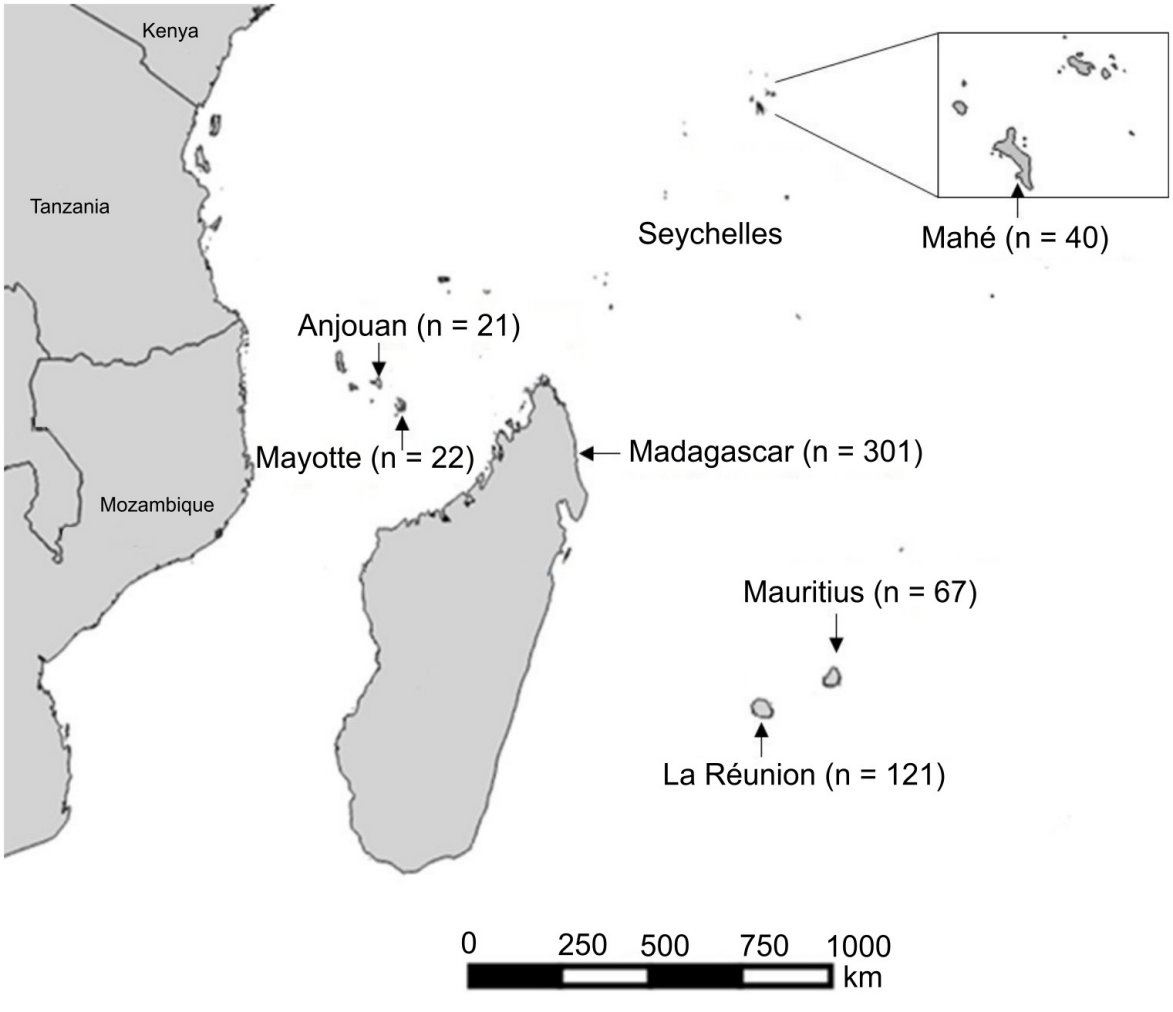
Geographic distribution of bats collected on the southwestern Indian Ocean islands of Anjouan (Union of Comoros), Mayotte (France), Madagascar, La Réunion (France), Mahé (Seychelles Archipelago) and Mauritius. n: number of bats sampled.

From March 2010 to March 2015, 653 bats representing 22 species from six families were collected from the islands of Anjouan and Mayotte (Comoros Archipelago), La Réunion and Mauritius (Mascarenes), Mahé (Seychelles Archipelago) and Madagascar (Fig 1). Both insectivorous and frugivorous bat species were captured using harp traps, hand nets and mist nets. Individual bats were identified by morphological characteristics [17] and in most cases by comparison to voucher specimens in The Field Museum of Natural History (FMNH) and The Université d’Antananarivo, Département de Biologie Animale (UADBA).

Animals were manipulated in accordance with the guidelines for the handling of wild mammals [18], and all field protocols were designed to strictly follow the terms of the research permits issued by national authorities in the different countries this study took place (S1 Text). Details on ethical clearance are provided (S2 Text). This study benefited from the sampling efforts conducted in the context of an ongoing long-term international project to catalogue the terrestrial vertebrate fauna of Madagascar [19]. The tissue samples collected during these inventories were optimized by different research programs and already reported on in different publications [20–25]. Bats trapped on Mayotte were released at the capture site after sampling. A total of 38 *Pteropus seychellensis* from Mahé and 12 *Pteropus rufus* from Madagascar, all destined for the bush meat trade, were obtained alive from local hunters, individual bats were processed within five minutes after death and tissue samples collected as for other islands (see below). Bat specimens sampled on the other islands were preserved as voucher specimens and curated in the FMNH and UADBA. Details concerning the sex and age (based on wing bone ossification and dental eruption patterns) [26] for each captured bat were recorded and are presented in database deposited on Dryad Digital Repository: http://dx.doi.org/10.5061/dryad.gp4h2. The names of capture sites were abbreviated (e.g. “Mangajou” as MGJ) and reported in S1 Table. An open-source Geographic Information System software, QGIS [27], was used to generate Fig 1.

Blood was collected either by veinipuncture of the brachial vein for bats that were released at the site of capture or by cardiac puncture for bats that were dispatched (in some cases, needles and syringes were rinsed with heparin or EDTA to prevent blood coagulation during the p rocedure). Sera were recovered by centrifugation at 3100 g for 5 min. Brain samples were collected from vouchered individuals. Tissue samples and sera were immediately stored in liquid nitrogen in the field and then transferred to -80°C freezers upon arrival at the laboratory.

### Detection of lyssavirus neutralising antibodies

Detection and titration of lyssavirus neutralising antibodies was performed using the miniaturised Rapid Fluorescent Focus Inhibition Test (RFFIT) [15]. The challenge viruses were the two most represented African bat associated lyssaviruses: DUVV (isolate DUVVSA06) and LBV (isolate LBVAFR1999) belonging to the phylogroups I and II, respectively. In some experiments, in order to delineate the antigenic relatedness of the putative locally circulating lyssavirus with other members of the *Lyssavirus* genus, sera were tested against two other representatives of phylogroup I: RABV (CVS-11) (n = 528) and EBLV-1 (isolate RV20) (n = 220).

Sera were heated at 56°C for 30 min to inactivate complement and tested at 1:5, 1:25, 1:125 and 1:625 dilutions. Each neutralisation assay was performed in triplicate and was read and validated by two different investigators. Serum samples producing significant viral neutralisation (i.e. > 50% reduction of fluorescent foci) at a dilution of 1:25 or higher, were considered positive for all tested viruses. Control wells were prepared with three dilutions of the challenge virus at 0.5FFD_50_, 5FFD_50_ and 50FFD_50_, and a well containing media and cell culture without virus was used as negative control. Mouse neuroblastoma cells at 2.10^6^ cells/ml were added to each well. Cultures were incubated in a humidified incubator at 37°C in the presence of 5% CO_2_ for 20 to 24 hours depending on the virus used. Slides were then acetone fixed and stained with 10 μl of polyclonal fluorescein isothiocyanate (FITC) conjugated immunoglobulin (Onderstepoort Veterinary Institute, South Africa) at a dilution of 1:300 with Evans Blue (0.5% in phosphate buffered saline (PBS)) for 30 min at 37°C. Slides were examined for fluorescent foci using an epifluorescence microscope. Results were validated if i) the negative control chamber (i.e. no virus) showed a confluent layer of cells and no fluorescent focus, ii) fluorescence foci were observed in the 0.5FFD_50_, 5FFD_50_ and 50FFD_50_ chambers (i.e. virus multiplication).

Of the 653 serum samples, 81 were excluded from analysis either because they displayed a cell-adhesion inhibitory-type cytotoxicity (n = 45) or because the volume of sera was insufficient to do the assay in triplicate (n = 36). *In finé*, the RFFIT could be performed for 572 sera. Results for each individual bat are presented in database deposited on Dryad Digital Repository: http://dx.doi.org/10.5061/dryad.gp4h2.

### Lyssaviruses RNA Detection

Although the Direct Fluorescent Antibody test (DFA) is the gold standard method to detect rabies antigen in brain tissue of rabid animals, we preferred to use the ultra-sensitive real time reverse transcription PCR, which can detect as low as 10 RNA copies, since all animals collected in our study were apparently “healthy” and we anticipated very low viral antigen expression in brain tissue [28]. Lyssavirus RNA detection was performed using a real time reverse transcription PCR (QuantiTect Probe RT-PCR Kit, QIAGEN, Cat. number 204443) with specific primers designed to target the conserved region of the nucleoprotein gene of lyssaviruses, including RABV, DUVV, EBLV-1 and EBLV-2 from phylogroup I, LBV and MOKV from phylogroup II [28].

### Statistical analysis

Statistical analyses were performed using Pearson chi-square (*χ*^2^) or Fisher’s exact tests in R software for calculation procedures (95% confidence intervals with a continuity correction). Probability values < 0.05 were considered statistically significant. All statistical computations were conducted using R version 3.0.0 [29].

## Results

A total of 572 sera from 421 insectivorous bats and 151 frugivorous bats belonging to six families and 22 species, were tested by RFFIT for neutralising antibodies against two African bat lyssaviruses namely DUVV and LBV representing phylogroups I and II, respectively. Sera from 42 individual bats out of 572 (7.3%) were capable of neutralising LBV and sera from 97 out of 540 (17.9%) were capable of neutralising DUVV. The cut off value for positivity was at 1:25 serum dilution but most bats seropositive for DUVV reacted at a dilution of 1:125 or higher (85.5% and 81.8% of sera from insectivorous and frugivorous bats, respectively (S1 Fig)). The same held true for bats seropositive for LBV, with 66.5% and 60.0% of sera from insectivorous and frugivorous bats, respectively, reacting at a dilution of 1:125 or higher (S1 Fig). Table 1 shows the levels of reactivity for the two challenge viruses segregated by island, bat family and species.

**Table 1:** Seroreactivity of bats from SWIO islands to lyssaviruses as assessed by RFFIT.

	Neutralisation of only one virus				Neutralisation of two viruses
	LBV	DUVV	EBLV-1	CVS-11	DUVV+EBLV-1
**Anjouan**	**2/21**	**2/8**	**0/0**	**0/8**	**0/0**
Miniopteridae					
*Miniopterus griveaudi*	0/2	0/1	0/0	0/8	0/0
Molossidae					
*Chaerephon pusillus*	2/19	2/7	0/0	0/0	0/0
**Madagascar**	**23/301**	**54/286**	**1/170**	**0/277**	**5/70**
Hipposideridae					
*Hipposideros commersoni*	0/6	1/6	1/4	0/6	1/4
Miniopteridae					
*Miniopterus* cf. *ambohitrensis*	0/17	4/15	0/4	0/15	0/4
*Miniopterus gleni*	0/4	0/4	0/0	0/4	0/0
*Miniopterus griveaudi*	2/33	10/29	0/6	0/23	0/6
*Miniopterus mahafaliensis*	0/11	0/11	0/0	0/9	0/0
*Miniopterus sororculus*	0/3	0/2	0/1	0/23	0/1
Molossidae					
*Chaerephon atsinanana*	0/16	0/15	0/0	0/16	0/0
*Chaerephon leucogaster*	0/28	9/28	0/6	0/26	1/6
*Mops leucostigma*	2/20	0/19	0/4	0/20	0/4
*Mops midas*	0/7	0/7	0/0	0/7	0/0
*Mormopterus jugularis*	3/58	17/58	0/8	0/58	0/8
*Otomops madagascariensis*	3/20	4/18	0/11	0/20	0/11
Pteropodidae					
*Eidolon dupreanum*	0/9	0/9	0/1	0/9	0/1
*Pteropus rufus*	0/12	5/11	0/5	0/10	3/5
*Rousettus madagascariensis*	7/35	4/33	0/16	0/33	0/16
Rhinonycteridae					
*Triaenops menamena*	2/11	0/11	0/2	0/7	0/2
Vespertilionidae					
*Myotis goudoti*	4/11	0/10	0/2	0/11	0/2
**Mahé (Seychelles)**	**4/40**	**6/40**	**1/17**	**0/40**	**1/17**
Pteropodidae					
*Pteropus seychellensis*	4/40	6/40	1/17	0/40	1/17
**Mauritius**	**7/67**	**19/67**	**2/29**	**0/62**	**0/29**
Molossidae					
*Mormopterus acetabulosus*	5/31	6/31	1/8	0/29	0/8
Pteropodidae					
*Pteropus niger*	2/36	13/36	1/21	0/33	0/21
**Mayotte**	**2/22**	**2/22**	**0/22**	**0/22**	**0/22**
Molossidae					
*Chaerephon pusillus*	1/3	1/3	0/3	0/3	0/3
Pteropodidae					
*Pteropus seychellensis*	2/19	1/19	0/19	0/19	0/19
**La Réunion**	**3/121**	**14/117**	**9/82**	**0/119**	**26/82**
Molossidae					
*Mormopterus francoismoutoui*	3/121	14/117	9/82	0/119	26/82
**Grand total**	**42/572**	**97/540**	**13/220**	**0/528**	**32/220**

Columns 2–5 show sera that neutralise LBV only, DUVV only, EBLV-1 only and CVS-11 only. Column six represents sera that cross neutralise both DUVV and EBLV-1 among the 220 sera tested against the two viruses. Fractions indicate at the numerator, the number of bats neutralising lyssaviruses and at the denominator the number of tested samples.

In order to delineate the antigenic relatedness of the putative lyssavirus that circulates in the SWIO region with other viruses of the genus, sera were tested against two other representatives of phylogroup I: RABV (CVS11) (n = 528) and EBLV-1 (isolate RV20) (n = 220). Of the 97 sera neutralising DUVV, 32 cross-neutralised EBLV-1 and not one cross-neutralised RABV. Of note, 13 sera out of 220 only neutralised EBLV-1 and not DUVV.

Bats seropositive to either of the two test viruses belonged to the six different bat families represented in our collections. Among the 22 bat species sampled from the different islands, four tested seropositive for DUVV only, three for LBV only and nine for DUVV and LBV (in different individuals). Six species were negative for all tested viruses. The seropositivity rates for DUVV and LBV did not differ significantly between islands, or between insectivorous and frugivorous species, or between males and females or between sub-adults and adults (p > 0.05). However, statistically significant differences exist between species and are described below.

Among the 14 insectivorous bat species studied on Madagascar, the highest seropositivity rate for DUVV was detected in *Miniopterus griveaudi* (34.5%; n = 10/29) (p < 0.001) and the highest seropositivity rate for LBV in *Myotis goudoti* (36.4%; n = 4/11) (p = 0.005). Of the three Malagasy frugivorous bat species, all endemic to the island, two (*Pteropus rufus* and *Rousettus madagascariensis*) tested seropositive for DUVV (45.5% n = 5/11; and 12.1%, n = 4/33; respectively), with the highest rates in *Pteropus rufus* (p = 0.01). Only *Rousettus madagascariensis* tested seropositive for LBV (20.0%; n = 7/35). The third frugivorous bat, *Eidolon dupreanum*, tested seronegative (n = 0/9) for LBV and DUVV.

Significant differences in seropositivity rates were recorded between the four bioclimatic zones and three altitudinal levels of Madagascar delineated herein, but only for DUVV. The highest seropositivity rates for DUVV were recorded in the sub-humid region (27.3%; n = 15/55) (p = 0.003) and in the highest elevation zone (29.5%; n = 13/44, p = 0.02). No significant difference was observed for seroreactivity for LBV among bioclimatic zones and altitudes. Significant differences were observed among localities (with rates above 50.0% of seropositivity for DUVV recorded in ANJOZ2 and BELO) (p < 0.001) but not between sites containing multiple species as compared to sites with single species nor between bat populations collected in caves, synanthropic roost sites or forests. Interestingly, bats belonging to the same species collected at different localities, displayed significantly different seropositivity rates. For example, *Miniopterus griveaudi* and *Rousettus madagascariensis* collected at multi-species sites (i.e. ANJHB) had significantly higher seropositivity rates for DUVV (75.0%) and LBV (40.0%), respectively, whereas significantly lower rates at monospecific sites (p < 0.05): in ANJHK1, 9.1% for DUVV in *Miniopterus griveaudi* (n = 1/11) and at ANJHB, 0.0% for LBV in *Rousettus madagascariensis* (n = 0/11) (p < 0.001). At the site level, specific patterns of lyssavirus neutralisation were recorded. At 21 positive sites on Madagascar, the neutralisation pattern appeared homogeneous among the seropositive bats at the specific site. In 11 localities bats seroneutralised DUVV only, at three localities bats cross-neutralised DUVV and EBLV-1 and at three localities bats seroneutralised LBV only. The four other positive localities combine animals, which expressed any of these three profiles of lyssavirus neutralisation (S2 Fig).

The reactivity profile of lyssavirus antibodies differed on the various regional islands (S3–S7 Figs) and, in most cases, positive sites combined different animals that neutralised either DUVV, EBLV-1, or cross neutralised DUVV and EBLV-1 and animals that neutralised LBV only. With the exception of Mayotte (S4 Fig), most sites on La Réunion (S5 Fig), Anjouan (S3 Fig), Mahé (S7 Fig) and Mauritius (S6 Fig), combined different animals that neutralised either phylogroup I viruses or LBV only.

In addition to the serology tests described above, brain samples from 550 of these individuals were screened for the presence of lyssavirus nucleic acid, all of which yielded negative results.

## Discussion

Islands of the southwestern Indian Ocean (SWIO) region, comprise one of the world’s most biodiverse hotspots [30], hosting a rich and unique chiropteran fauna. For example, on Madagascar, of the 45 recognized bat species, 36 are endemic to the island [31, 32]; the neighbouring SWIO islands have notably lower measures of bat species diversity [19]. Several studies have reported on seroprevalence to lyssaviruses in bats from Africa and Asia [16, 33–39], but until now limited information was available for SWIO islands, located between these two continents. As compared to continents, island ecosystems have specificities and peculiarities that may influence the epidemiology of infection in humans and animals [40]. Indeed, our study reveals serological evidence of wide spread lyssaviruses exposure among frugivorous and insectivorous bats on these islands (including those deemed rabies free). At a regional scale, our study considerably expands the geographic coverage and bat species investigated on six different islands, when compared to the single study previously published on two Malagasy fruit bats, *Eidolon dupreanum* and *Pteropus rufus* [14].

Our data reveal that on SWIO islands, antibodies to lyssaviruses are present at high rates, both in frugivorous and insectivorous bats as already reported from Asia [37, 38]. The rates of seropositivity reported in our study varied from 10.0% to 44.4% in bat species for which the sampling was sufficient (n > 20 individuals). The high positive rates reported herein have also been found in other studies outside the region of insectivorous bat species, such as 60.8% (n = 45/74) for *Taphozous* sp. and 50.0% (n = 63/126) for *Hipposideros larvatus* in northern Vietnam [37], 59.0% (n = 62/105) for *Scotophilus kuhlii* and 54.1% for *Philetor brachypterus* in the Philippines [39]. Similarly, high rates have been reported in frugivorous bat species, such as 51.9% (n = 14/27) for *Pteropus hypomelanus* in the Philippines [39].

Although the high detection rates of lyssavirus infection in some bat species may indicate that they are particularly susceptible to virus transmission, prevalence data should be interpreted with prudence; they represent only a momentary picture of the immunity to *Lyssavirus* in the study sample at the specific moment they were collected and in some cases based on a limited number of individuals. Seroprevalence levels may change over time on a given island and across bat species, which may depend on age and sex, antibody kinetics, individual B cell memory responses, co-infections, seasonal dynamics of infection, etc. These aspects clearly indicate caution to not over interpret the quantitative data, specifically in the absence of comparative analysis of longitudinal studies that are complicated to conduct in wild animal populations. Our results revealed significant differences related to bat species, roost sites and bioclimatic regions on Madagascar, and further suggest the role of abiotic or biotic factors (e.g. climate, altitude, sex, etc.) in lyssavirus circulation and dynamics as previously reported for Malagasy bats and their hosted paramyxoviruses [25].

Our data highlight that 12 insectivorous bat species and three out of the four frugivorous bat species from the SWIO region were exposed to lyssaviruses related to or similar to DUVV or LBV. Moreover, we recorded antibodies reacting to DUVV or LBV in nine bat species indicating an unrestricted circulation of these two viral species. Based on the sero-neutralisation profiles, at least one circulating *Lyssavirus* appears referable to phylogroup I, since most of the positive sera were able to neutralise DUVV or EBLV-1. In fact, at the regional level, the highest rates of seroreactivity were recorded against DUVV (17.9%), followed by a dual reactivity with DUVV and EBLV (14.5%), and a lower rate with LBV (7.3%). This reactivity profile is conserved across the different island populations tested in this study.

These results also support the view that a phylogroup II lyssavirus, closely related to, if not LBV itself, is circulating in the region. This is in agreement with the study of Reynes and colleagues [14], who reported that 24% of sera from Malagasy *Eidolon dupreanum* neutralised only LBV. In our series, the highest seropositivity rates against LBV across the different island and bat species were recorded on Mayotte and Mauritius (13.6% and 10.4%, respectively) and in populations of *Myotis goudoti* and *Rousettus madagascariensis* on Madagascar (36.4% and 20.0%, respectively).

The use of EBLV-1 and Rabies lyssavirus (CVS-11) as additional challenge viruses in the RFFIT, allowed delineation of cross reactivity profiles (Table 1). The observation that bat sera neutralizing DUVV occasionally cross neutralise EBLV-1 was expected, as the two viruses belong to the same phylogroup I. However, it strikingly contrasts with the absence of any cross neutralisation with CVS-11 (RABV), which also belongs to the same phylogroup [4, 33]. Cross neutralisation between lyssaviruses, within either phylogroup I or II has been previously reported [4, 41]. Experimental studies in animals also supported cross protection induced by RABV vaccines to other lyssaviruses of phylogroup I [42–45]. The lyssavirus glycoprotein carries most of the antigenic epitopes and regions targeted by neutralising antibodies [46]. Antibodies against conserved G protein epitopes shared by two lyssaviruses may allow cross neutralisation of these two lyssaviruses [4].

A brief comment is needed to explain why bat sera neutralizing DUVV/EBLV1 do not cross neutralize CVS11, even though they all belong to the same phylogroup. Cross–antigenicity is best detected by hyperimmune antisera generated after separate active immunization of mice or rabbits for each virus [4]. It is not required that a natural infection (such as in bats naturally infected by *Lyssavirus*) should constantly elicit antibodies specific for cross-antigenic epitopes, even if the latter do exist. This will depend on their intrinsic antigenicity and the strength of the immune antibody response after natural infection. Moreover, even if such antibodies were generated at some point after natural infection, they may have subsequently disappeared, due to their own kinetics compared to that of non-cross neutralizing antibodies. Antibodies to *Lyssavirus* detected in bat sera, persisting long after remote infection, most likely represent only a fraction of the repertoire of specific memory B cells.

Thus, the profile of cross neutralisation recorded in our study may indicate closer antigenic relativity of the putative lyssavirus(es) circulating in the SWIO with DUVV and EBLV-1 than with RABV. This might be particularly true in an insectivorous bat species from La Réunion, *Mormopterus francoismoutoui*, for which high rates of reactivity for EBLV-1 only and cross reactivity with DUVV and EBLV-1 were detected.

Madagascar and islands making up the Comoros archipelago are closer to the African continent than the more outlying islands of the Mascarenes and those sampled in the Seychelles archipelago. Among the lyssaviruses belonging to phylogroups I and II, only DUVV, SHIV and LBV, have been isolated to date from African bats. Hence, DUVV and LBV might be the putative lyssaviruses actually circulating on SWIO islands. Alternatively, the latter might correspond to closely related and unidentified new lyssavirus species. However, this will require successful isolation of these viruses and further molecular and genetic characterisation for their detailed diagnosis.

Although the main aim of this study was to detect serologic traces of exposure to *Lyssavirus* amongst bats of the SWIO islands [16], we also tested available brain samples for the presence of lyssavirus nucleic acids. We did not detect lyssavirus RNA in the brain of any insectivorous or frugivorous bat. This is not an unexpected result as no abnormal morbidity or mortality among sampled bats was observed during our study. This result indicates that the potential risk for humans or other animals to be exposed to an actively infected bat is low; however, the serological evidence indicates that rabies related lyssaviruses do circulate in bat populations in the SWIO. In the Old World, very few cases of human rabies due to rabies-related bat lyssaviruses are known: to date three fatal cases have been reported for DUVV [47], whilst no human case due to LBV has been confirmed. However, cases of rabies in Africa and Asia are known to be under estimated and when reported, the causative lyssavirus is seldom characterized. In the SWIO region, an increase awareness of the risk of rabies transmission is recommended after contact with bats, specifically via biting or scratching or saliva contact on open wounds or mucosal membranes. Any exposure should be immediately followed by appropriate post exposure prophylactic actions as recommended by the WHO guidelines [48].

## Supporting Information

S1 Fig Distribution of neutralising antibody titers to DUVV and LBV in bats from southwestern Indian Ocean islands as assessed by the Rapid Fluorescent Focus Inhibition Test. Results represent the proportion of sera from insectivorous and frugivorous bat species titering at 1/25 (the cut off value of positivity), 1/125, and 1/625 among the sera tested positive for each challenge virus.
(TIF)

S2 Fig Sites recorded with samples seropositive to lyssaviruses and lyssaviruses neutralisation profiles on Madagascar. Abbreviations next to illustrated islands indicate names of capture sites (e.g. ANKPK for “Ankapoka”) reported in S1 Table. Coloured site names and in the chart correspond to the lyssaviruses antibodies detected in bats: sera neutralising DUVV only are in blue; those neutralising LBV only are in red, those cross-neutralising DUVV + EBLV-1 are in yellow. Sites which combine animals which sera neutralised DUVV only, EBLV-1 only, cross-neutralised DUVV and EBLV-1 and animals neutralised LBV only are in brown. The six red squares indicate the provincial capitals.
(TIF)

S3 Fig Sites recorded with samples seropositive to lyssaviruses and lyssaviruses neutralisation profiles on Anjouan. Abbreviations next to illustrated islands indicate names of capture sites (e.g. COA for “College d'Ouani”) reported in S1 Table. Coloured site names and in the chart correspond to the lyssaviruses antibodies detected in bats: sera neutralising DUVV only are in blue and sites which combine animals which sera neutralised DUVV only and LBV only are in brown.
(TIF)

S4 Fig Sites recorded with samples seropositive to lyssaviruses and lyssaviruses neutralisation profiles on Mayotte. Abbreviations next to illustrated islands indicate names of capture sites (e.g. MGJ for “Mangajou”) reported in S1 Table. Coloured site names and in the chart correspond to the lyssaviruses antibodies detected in bats: sera neutralising DUVV only are in blue and those neutralising LBV only are in red.
(TIF)

S5 Fig Sites recorded with samples seropositive to lyssaviruses and lyssaviruses neutralisation profiles on La Réunion. Abbreviations next to illustrated islands indicate names of capture sites (e.g. GTB for “Grotte de Trois Bassin”) reported in S1 Table. Coloured site names and in the chart correspond to the lyssaviruses antibodies detected in bats: sera neutralising DUVV only are in blue and sites which combine animals which sera cross-neutralised DUVV+ EBLV-1 or LBV only are in brown.
(TIF)

S6 Fig Sites recorded with samples seropositive to lyssaviruses and lyssaviruses neutralisation profiles on Mauritius. Abbreviations next to illustrated islands indicate names of capture sites (e.g. CSC for “Cascavelle”) reported in S1 Table. Coloured site names and in the chart correspond to the lyssaviruses antibodies detected in bats: sera neutralising DUVV only are in blue; those cross-neutralising DUVV+EBLV-1 are in yellow. Sites which combine animals which sera neutralised DUVV only or EBLV-1 only and animals neutralised LBV only are in brown.
(TIF)

S7 Fig Sites recorded with samples seropositive to lyssaviruses and lyssaviruses neutralisation profiles on Mahé. Abbreviations next to illustrated islands indicate names of capture sites (e.g. FRL for “FairyLand”) reported in S1 Table. Coloured site names and in the chart correspond to the lyssaviruses antibodies detected in bats: sera neutralising. Sites which combine animals which sera neutralised DUVV only, EBLV-1 only, cross-neutralised DUVV+EBLV-1 and animals neutralised LBV only are in brown.
(TIF)

S1 Table **Information from sites on Anjouan, Madagascar, Mahé, Mauritius, Mayotte and La Réunion,** indicated as: the island name, the site name (and abbreviation), GPS coordinates, type of habitat and number of species recorded at each site (one or more than one).
(DOC)

S1 Text Authorisations for sampling of bats on different southwestern Indian Ocean islands. List of authorisations required for bat sampling from Madagascar, La Réunion, Mayotte, Anjouan, Mahé and Mauritius authorities.
(DOC)

S2 Text Ethical clearance information. Ethical procedure applied for bat sampling on Madagascar, La Réunion, Mayotte, Anjouan, Mahé and Mauritius.
(DOCX)
