# Supplementary figures and images for: Serological Evidence of Lyssaviruses among Bats on Southwestern Indian Ocean Islands
Source: PLoS One. 2016 Aug 8;11(8):e0160553. doi: 10.1371/journal.pone.0160553 (PMC4976896; doi:10.1371/journal.pone.0160553)

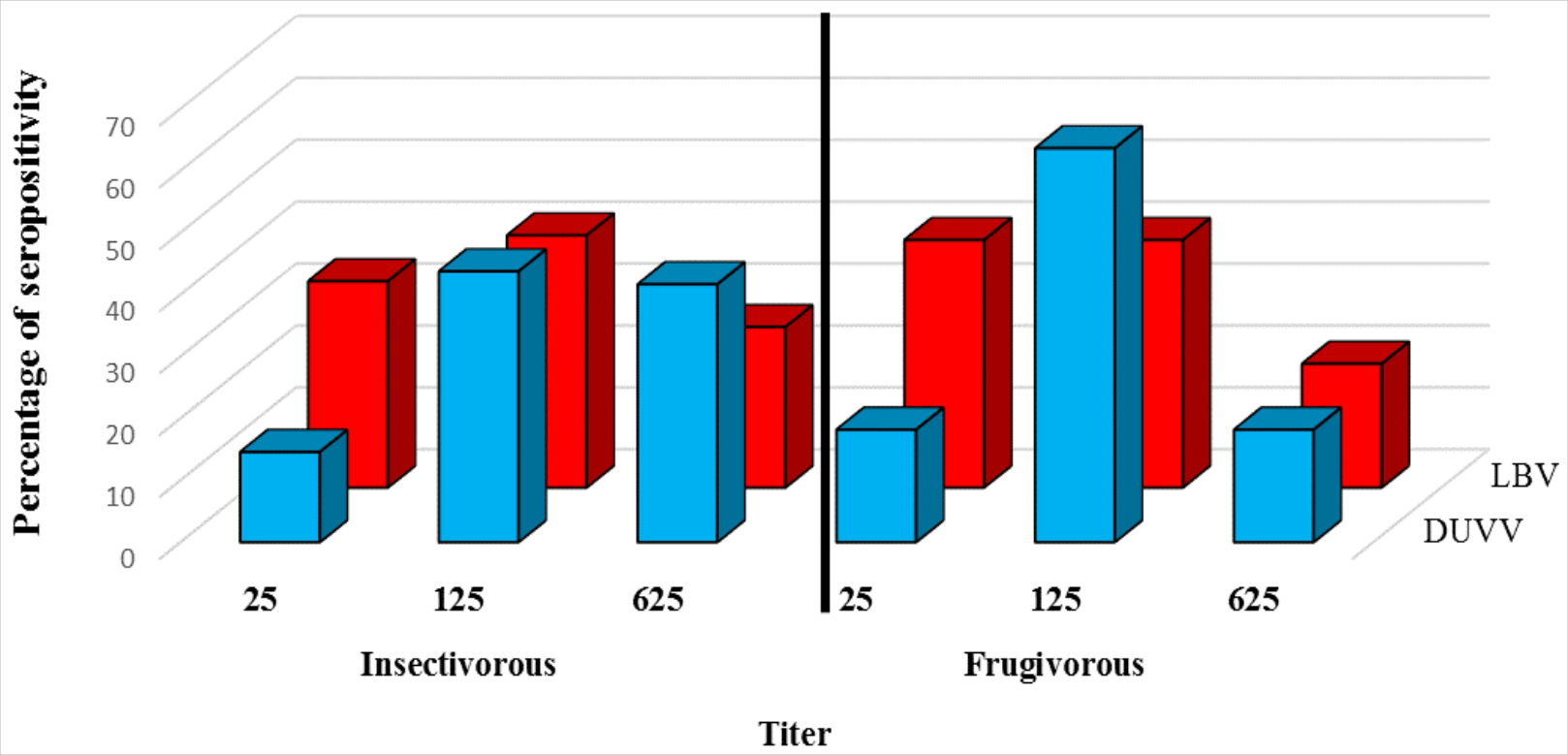

Supplement: S1 Fig — Results represent the proportion of sera from insectivorous and frugivorous bat species titering at 1/25 (the cut off value of positivity), 1/125, and 1/625 among the sera tested positive for each challenge virus. (TIF) [file pone.0160553.s001.tif]

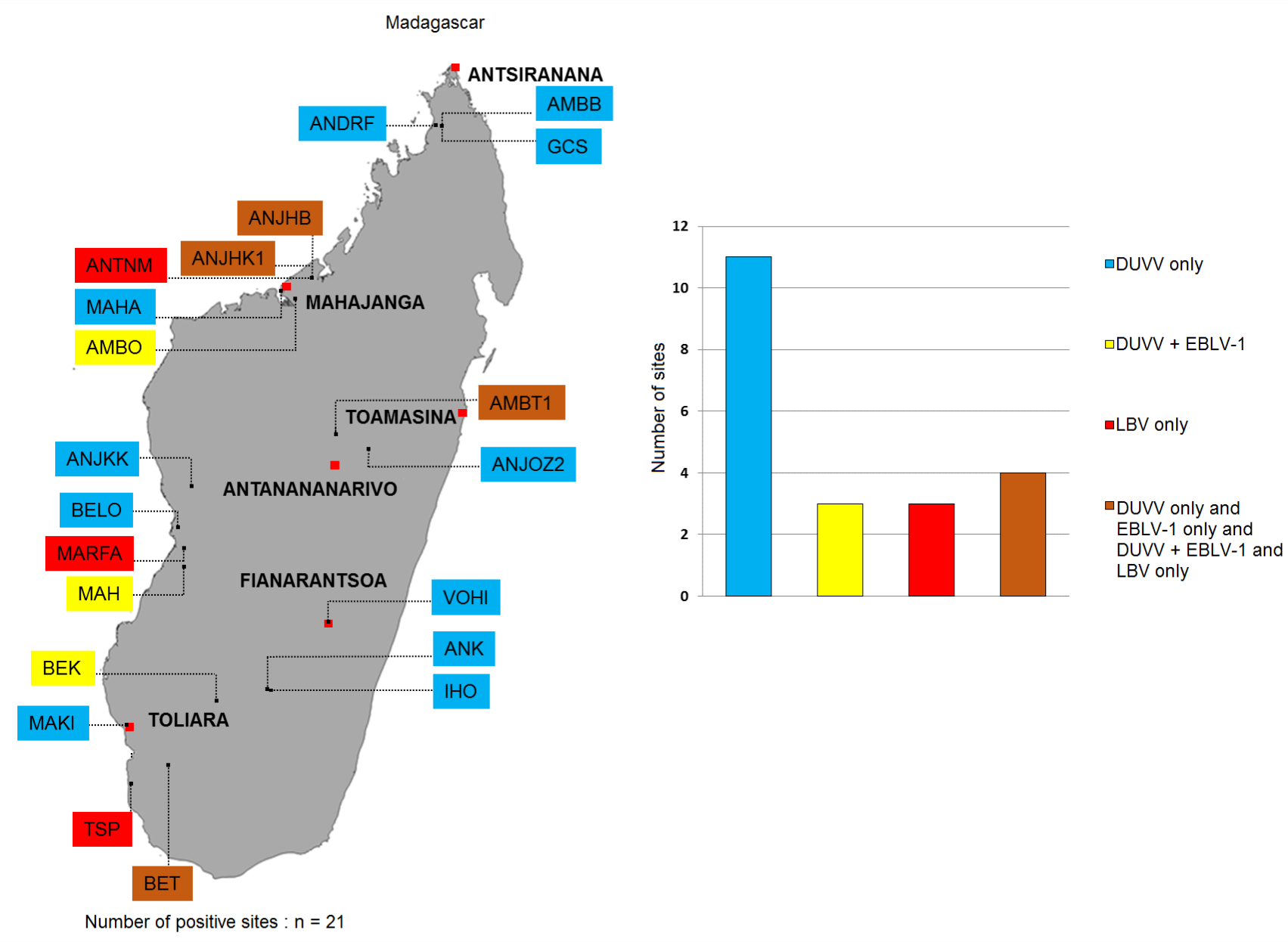

Supplement: S2 Fig — Abbreviations next to illustrated islands indicate names of capture sites (e.g. ANKPK for “Ankapoka”) reported in S1 Table. Coloured site names and in the chart correspond to the lyssaviruses antibodies detected in bats: sera neutralising DUVV only are in blue; those neutralising LBV only are in red, those cross-neutralising DUVV + EBLV-1 are in yellow. Sites which combine animals which sera neutralised DUVV only, EBLV-1 only, cross-neutralised DUVV and EBLV-1 and animals neutralised LBV only are in brown. The six red squares indicate the provincial capitals. (TIF) [file pone.0160553.s002.tif]

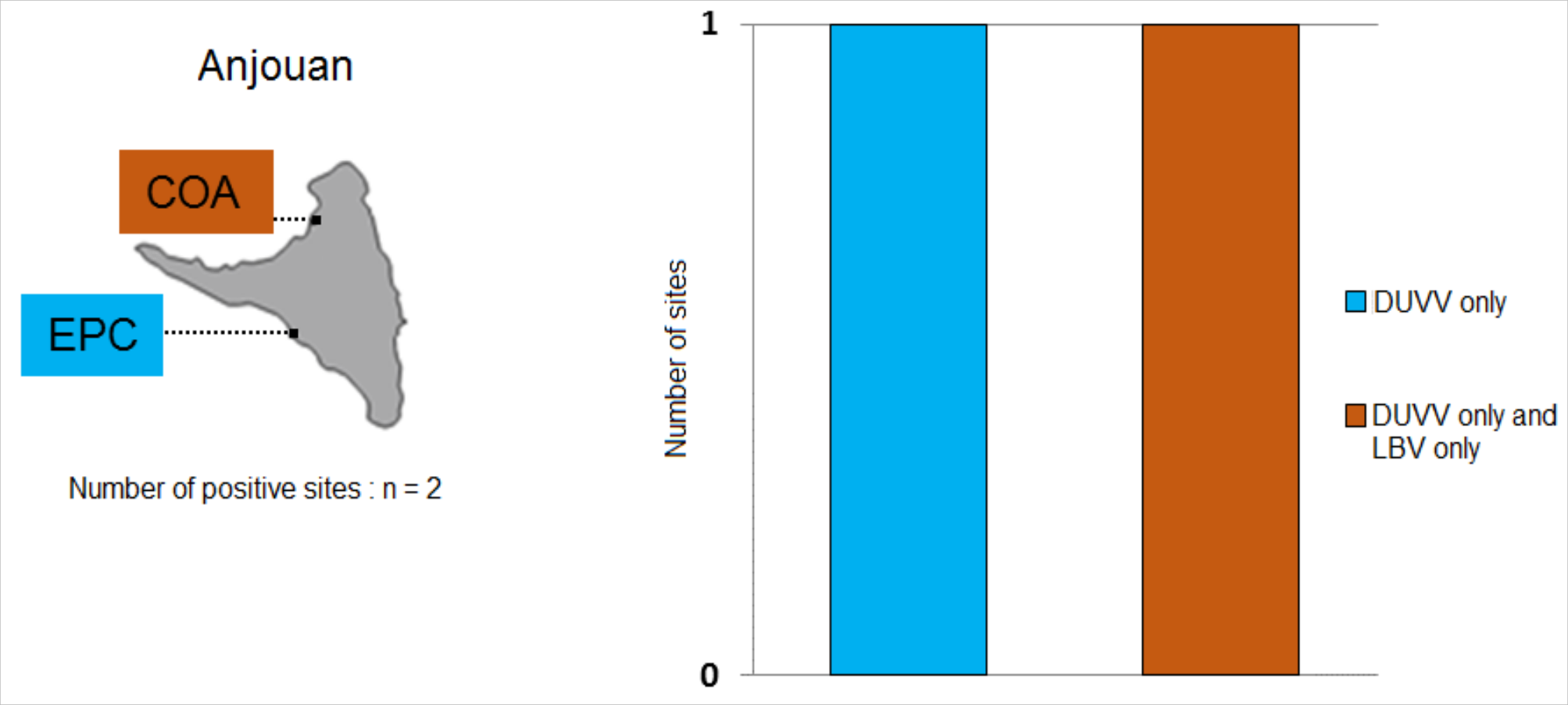

Supplement: S3 Fig — Abbreviations next to illustrated islands indicate names of capture sites (e.g. COA for “College d'Ouani”) reported in S1 Table. Coloured site names and in the chart correspond to the lyssaviruses antibodies detected in bats: sera neutralising DUVV only are in blue and sites which combine animals which sera neutralised DUVV only and LBV only are in brown. (TIF) [file pone.0160553.s003.tif]

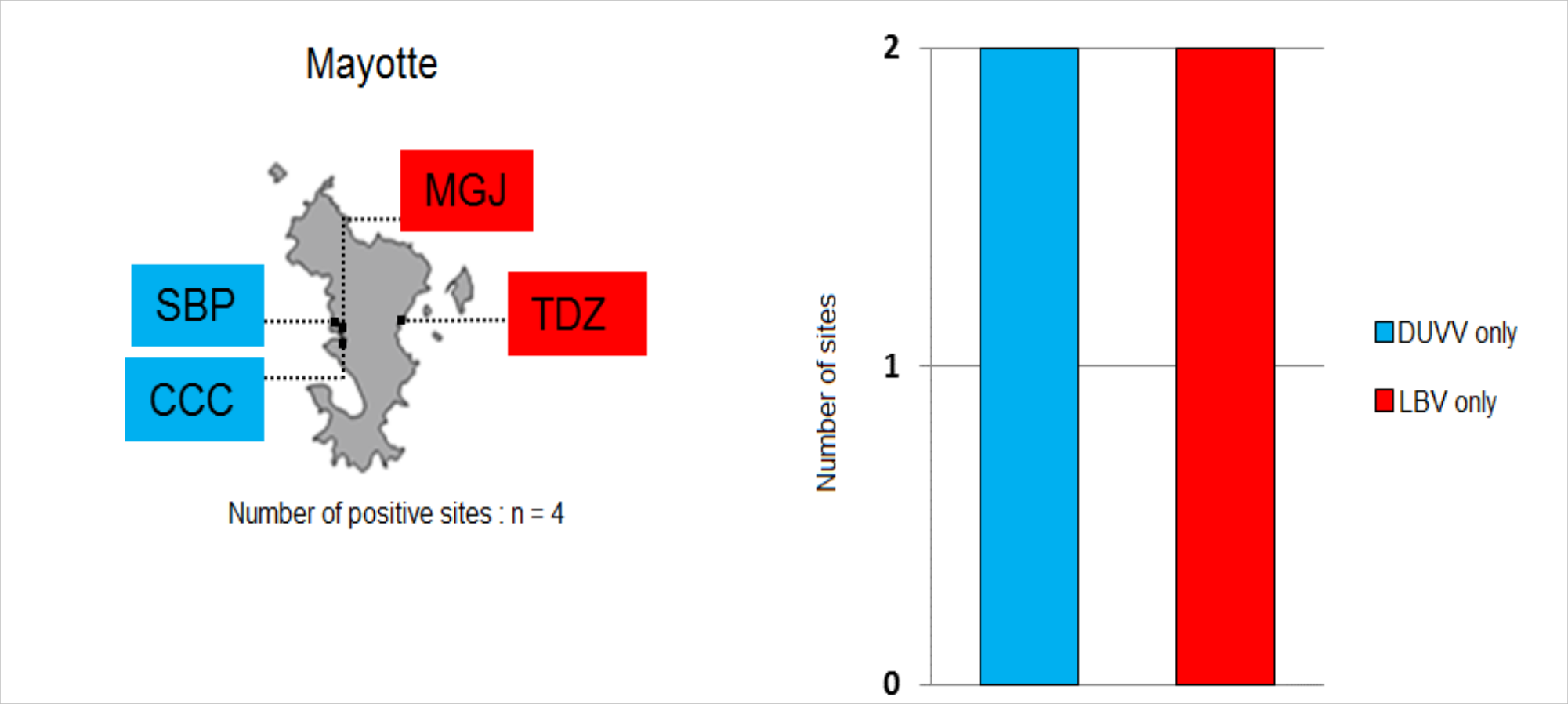

Supplement: S4 Fig — Abbreviations next to illustrated islands indicate names of capture sites (e.g. MGJ for “Mangajou”) reported in S1 Table. Coloured site names and in the chart correspond to the lyssaviruses antibodies detected in bats: sera neutralising DUVV only are in blue and those neutralising LBV only are in red. (TIF) [file pone.0160553.s004.tif]

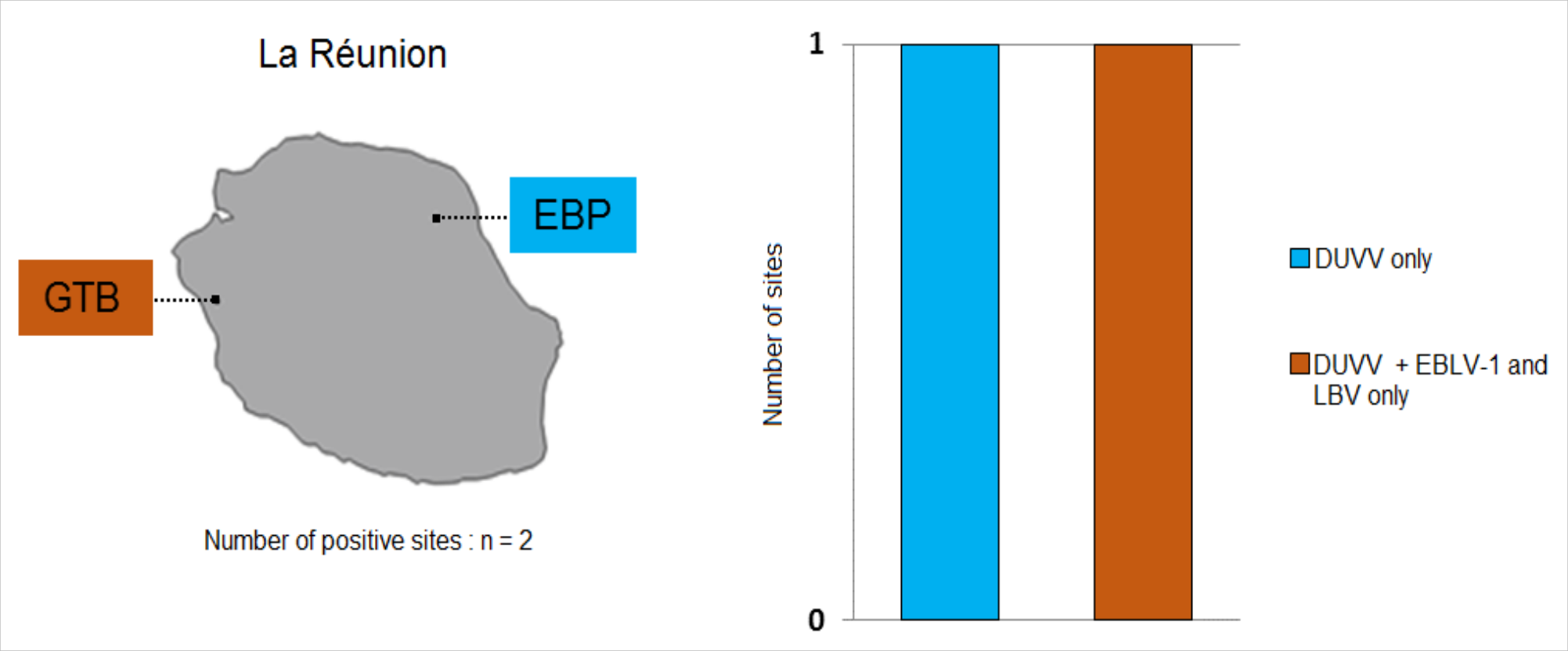

Supplement: S5 Fig — Abbreviations next to illustrated islands indicate names of capture sites (e.g. GTB for “Grotte de Trois Bassin”) reported in S1 Table. Coloured site names and in the chart correspond to the lyssaviruses antibodies detected in bats: sera neutralising DUVV only are in blue and sites which combine animals which sera cross-neutralised DUVV+ EBLV-1 or LBV only are in brown. (TIF) [file pone.0160553.s005.tif]

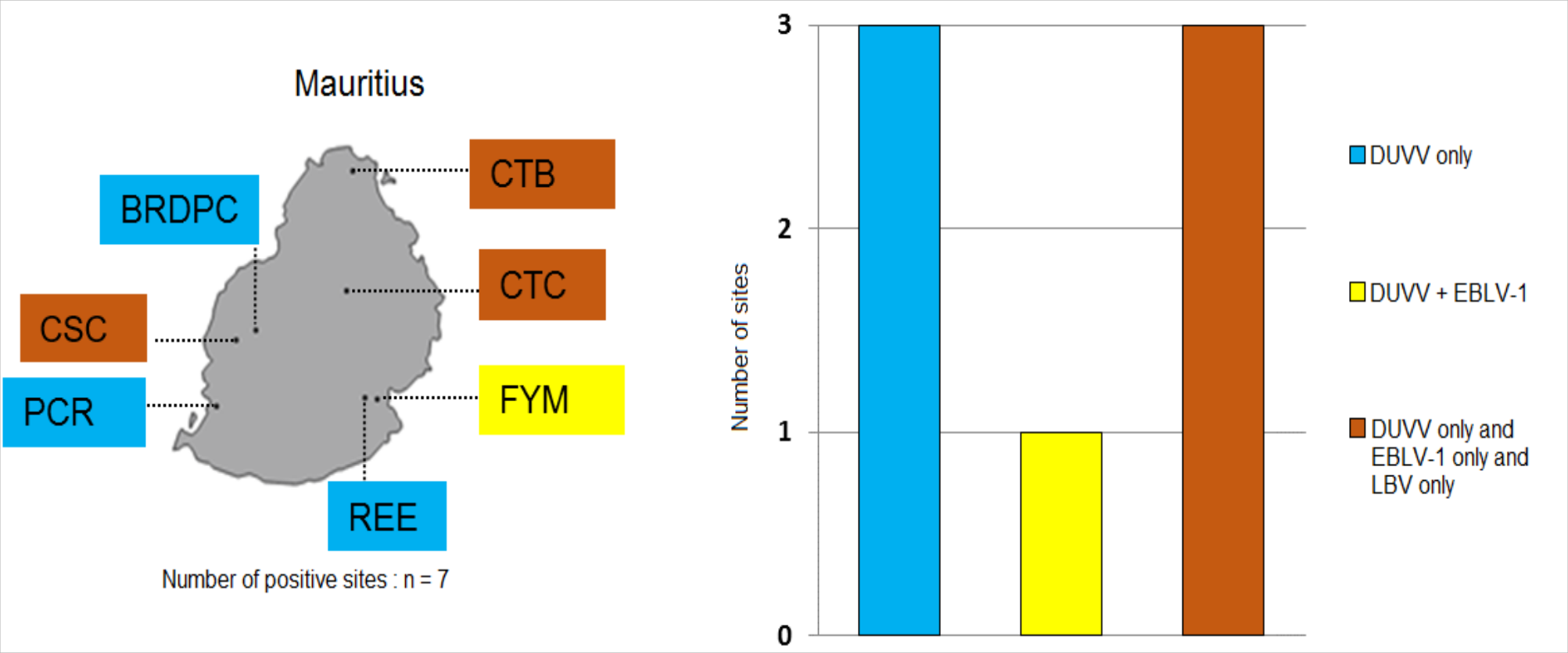

Supplement: S6 Fig — Abbreviations next to illustrated islands indicate names of capture sites (e.g. CSC for “Cascavelle”) reported in S1 Table. Coloured site names and in the chart correspond to the lyssaviruses antibodies detected in bats: sera neutralising DUVV only are in blue; those cross-neutralising DUVV+EBLV-1 are in yellow. Sites which combine animals which sera neutralised DUVV only or EBLV-1 only and animals neutralised LBV only are in brown. (TIF) [file pone.0160553.s006.tif]

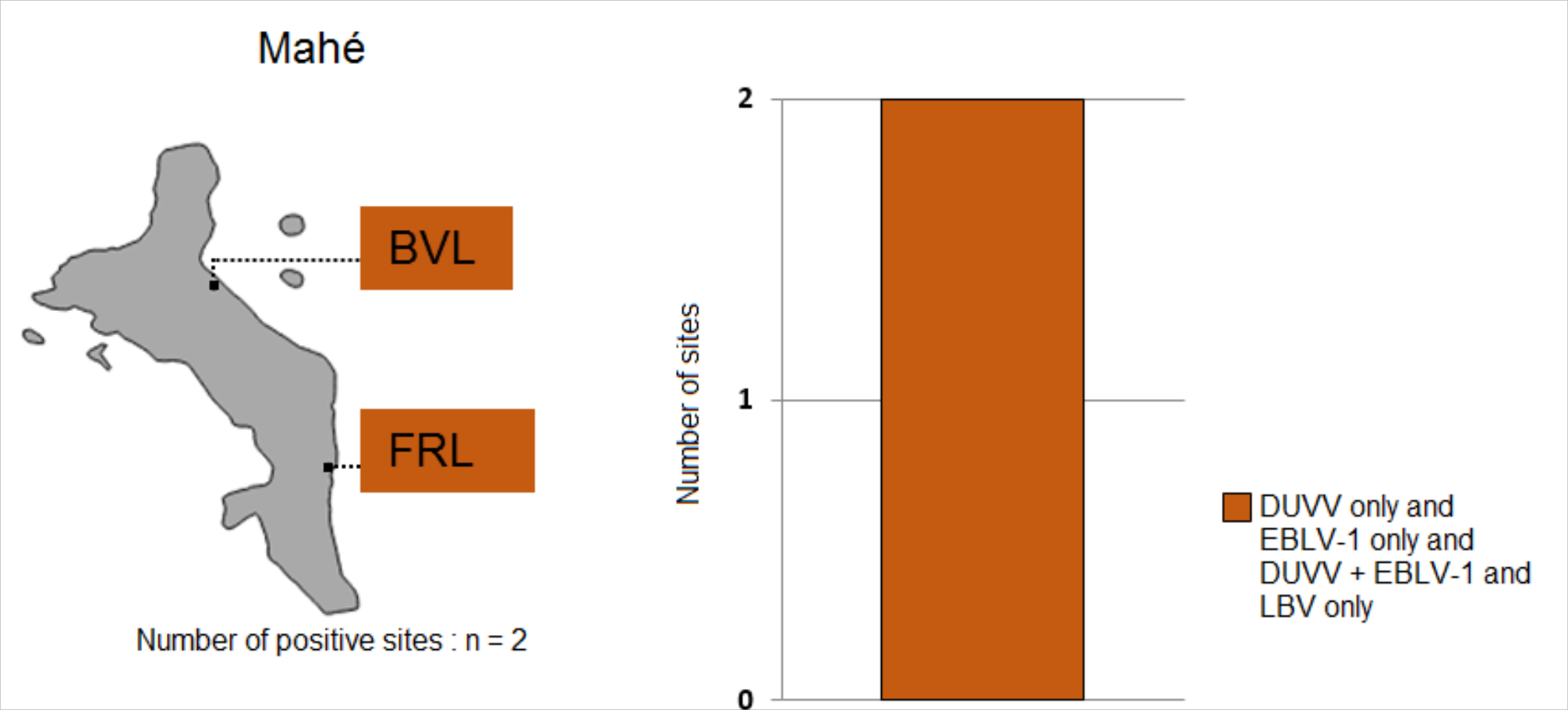

Supplement: S7 Fig — Abbreviations next to illustrated islands indicate names of capture sites (e.g. FRL for “FairyLand”) reported in S1 Table. Coloured site names and in the chart correspond to the lyssaviruses antibodies detected in bats: sera neutralising. Sites which combine animals which sera neutralised DUVV only, EBLV-1 only, cross-neutralised DUVV+EBLV-1 and animals neutralised LBV only are in brown. (TIF) [file pone.0160553.s007.tif]
