# Supplementary material for: Serological Evidence of Lyssaviruses among Bats on Southwestern Indian Ocean Islands
Source: PLoS One. 2016 Aug 8;11(8):e0160553. doi: 10.1371/journal.pone.0160553 (PMC4976896; doi:10.1371/journal.pone.0160553)
Supplement: S1 Table — Information from sites on Anjouan, Madagascar, Mahé, Mauritius, Mayotte and La Réunion, indicated as: the island name, the site name (and abbreviation), GPS coordinates, type of habitat and number of species recorded at each site (one or more than one). (DOC) [file pone.0160553.s008.doc]

**S1 Table.** Information from sites on Anjouan, Madagascar, Mahé, Mauritius, Mayotte and La Réunion, indicated as: the island name, the site name (and abbreviation), GPS coordinates, type of habitat and number of species recorded at each site (one or more than one).

| **Islands** | **Abbreviations** | **Site names** | **Latitude** | **Longitude** | **Type of habitat** | **Species number** |
| --- | --- | --- | --- | --- | --- | --- |
| Anjouan | GBK | Grotte de Buemokolo | -12.1807 | 44.4461 | Cave | 1 |
| Anjouan | COA | College d'Ouani | -12.1355 | 44.42422 | Building | 1 |
| Anjouan | EPC | Ecole primaire de Chirove | -12.2708 | 44.3978 | Building | 1 |
| Madagascar | TSP | Parc National de Tsimanampetsotsa, Grotte d'Andranoilovy | -24.05000 | 43.75000 | Cave | >1 |
| Madagascar | VINT | Grotte de Vintane, (Vintany) 4.1 km SE Itampolo | -24.70236 | 43.96372 | Cave | >1 |
| Madagascar | CGS | Parc National d'Ankarana, Grotte des chauves-souris, 3 km NW Mahamasina | -12.956317 | 49.11808 | Cave | 1 |
| Madagascar | ANJKK | Parc National de Bemaraha, Anjohikinakina 15.5 km N Bekopaka | -19.009900 | 44.76770 | Cave | >1 |
| Madagascar | ANJHB | Grotte d'Anjohibe-3.7 km NE Antanamarina | -15.538150 | 46.88598 | Cave | >1 |
| Madagascar | ANTNM | Cascade d'Antanamarina | -15.576183 | 46.86928 | Forestry | >1 |
| Madagascar | ANKPK | Ankapoka | -19.04490 | 44.77350 | Forestry | 1 |
| Madagascar | AMBO | Ambovondramanesy village near Berivotra, along RN4 | -15.900000 | 46.58330 | Building | 1 |
| Madagascar | TANA | Grotte de Tanambao (Bishiko) 0.75 km E St Augustin | -23.54888 | 43.76740 | Cave | >1 |
| Madagascar | AMBB | Parc National d'Ankarana, Ambahibe Cave, 2 km W. Mahamasina | -12.967667 | 49.12052 | Cave | >1 |
| Madagascar | AMBT | Réserve Spéciale d'Ambohitantely, Grotte des chauve-souris | -18.181167 | 47.28930 | Cave | >1 |
| Madagascar | AMBT2 | Réserve Spéciale d'Ambohitantely, Grotte des chauve-souris | -18.181167 | 47.28930 | Forestry | 1 |
| Madagascar | MAKI | Grotte de Makis (Mikea), near Hotel la Mangrove on Toliara-St Augustin Road | -23.47211 | 43.77069 | Cave | >1 |
| Madagascar | ANK | Commune rurale d'Ankily, west of Ihosy off RN7 | -22.385000 | 46.09556 | Building | 1 |
| Madagascar | BET | Betioky Sud, New Lutherian church | -23.71986 | 44.38350 | Building | 1 |
| Madagascar | IHO | Ihosy, Bureau du chef de la Région | -22.403360 | 46.12887 | Building | 1 |
| Madagascar | VOHI | Vohipoa, CSB II | -20.995980 | 47.16156 | Building | >1 |
| Madagascar | ANJOZ2 | Ambohibeloma, 3,2km W. Anjozorobe | -18.408820 | 47.85168 | Forestry | 1 |
| Madagascar | ANDA1 | CEG Andasibe | -18.92016 | 48.41768 | Building | >1 |
| Madagascar | SAK1 | Sakaraha, Direction des Eaux et forêt, Bureau chef de cantonnement | -22.90946 | 44.52279 | Building | 1 |
| Madagascar | TSMIF | Tsimafana, CEG de Tsimafana | -19.72350 | 44.58432 | Building | >1 |

| **Islands** | **Abbreviations** | **Site names** | **Latitude** | **Longitude** | **Type of habitat** | **Species number** |
| --- | --- | --- | --- | --- | --- | --- |
| Madagascar | MARFA | Marofandilia, Ecole primaire | -20.06743 | 44.65800 | Building | 1 |
| Madagascar | ANTAN | Antanandava, Eglise FLM 5.8 km NE Beroboka Sud | -19.92973 | 44.60594 | Building | 1 |
| Madagascar | BEK | Parc National d'Isalo, Grotte de Bekapity | -22.633336 | 45.21808 | Cave | >1 |
| Madagascar | AMB | Grotte d'Ambanilia 3.7 km SSE Sarodrano | -23.53995 | 43.74605 | Cave | 1 |
| Madagascar | ANJHK1 | Grotte d'Anjohikely (south entrance), 1.5 km NE Antanamarina | -15.560883 | 46.87420 | Cave | 1 |
| Madagascar | ANDRF | Parc National d'Ankarana, Grotte d'Andrafiabe, 3.3 ESE Andrafiabe | -12.931667 | 49.06000 | Cave | >1 |
| Madagascar | CROCO | Parc National d'Ankarana, 2,2 km ESE Amboandriky, Grotte d'Ambatoharanana, (Crocodile Cave) | -12.988300 | 49.02170 | Cave | >1 |
| Madagascar | ISALO1 | Parc National d'Isalo,3.8 km NW de Ranohira, along Namaza river | -22.540000 | 45.38000 | Forestry | >1 |
| Madagascar | ANDOK | Parc National d'Ankarana, Grotte du troisiéme Canyon, along Andokotokana River | -12.914170 | 49.05500 | Cave | 1 |
| Madagascar | MAHA | North of Mahajanga, petite plage | -15.667970 | 46.32205 | Forestry | 1 |
| Madagascar | BELO | Belo Tsiribihina, central hospital | -19.70040 | 44.54750 | Building | >1 |
| Madagascar | MAH | Mahabo, EPP de Mahabo | -20.37770 | 44.66072 | Building | >1 |
| Mahé | FRL | FairyLand | -4.730203 | 55.520 | Forestry | 1 |
| Mahé | BVL | BeauVallon | -4.63639 | 55.460 | Building | 1 |
| Mauritius | CSC | Cascavelle | -20.29532 | 57.41922 | Forestry | 1 |
| Mauritius | PCR | Petite Case Noyale | -20.40083 | 57.38332 | Forestry | 1 |
| Mauritius | REE | Riche en Eau | -20.38797 | 57.65424 | Forestry | 1 |
| Mauritius | FYM | Fayence Moutain | -20.38925 | 57.67780 | Forestry | 1 |
| Mauritius | BRDPC | Black River District, Palma Cave | -20.27778 | 57.45389 | Cave | 1 |
| Mauritius | CTC | Camp Thorel Cave | 20.21559 | 57.62042 | Cave | 1 |
| Mauritius | CTB | Caverne Trois Bras | -20.02389 | 57.63306 | Cave | 1 |
| Mayotte | SBP | Sohoa be plage | -12.822778 | 45.10638 | Forestry | >1 |
| Mayotte | MGJ | Mangajou | -12.8435847 | 45.11640 | Forestry | 1 |
| Mayotte | CCC | Carrefour Chiconi | -12.8331 | 45.11439 | Forestry | >1 |
| Mayotte | TDZ | Tsoundzou 2 | -12.818479 | 45.1974239 | Forestry | 1 |
| La Réunion | GTB | Grotte de Trois Bassin | -21.103838 | 55.292083 | Cave | 1 |
| La Réunion | EBP | Eglise de Bras-Panon | 20.98112 | 55.64301 | Building | 1 |
