# Supplementary material for: Serological Evidence of Lyssaviruses among Bats on Southwestern Indian Ocean Islands
Source: PLoS One. 2016 Aug 8;11(8):e0160553. doi: 10.1371/journal.pone.0160553 (PMC4976896; doi:10.1371/journal.pone.0160553)
Supplement: S1 Text — List of authorisations required for bat sampling from Madagascar, La Réunion, Mayotte, Anjouan, Mahé and Mauritius authorities. (DOC) [file pone.0160553.s009.doc]

**S1 Text**. Authorisations for sampling of bats on different Southwestern Indian Ocean islands

1. Madagascar: Direction du Système des Aires Protégées and Direction Générale de l’Environnement et des Forêts; Madagascar National Parks: Export permit n°194/12/MEF/SG/DGF/DCB.SAP/SCB, 067/12/MEF/SG/DGF/DCB.SAP/SCBSE, and 032/12/MEF/SG/DGF/DCB.SAP/SCBSE. A CITES permit from the Malagasy national authority was issued for tissue export (permit 243C-EA06/MG12) to CRVOI on Reunion.
2. La Réunion: Préfecture de La Réunion: Arrêté préfectoral of 11 Février 2013.
3. Mayotte: Préfecture de Mayotte: Arrêté préfectoral n°158/DEAL/SEPR/2014.
4. Anjouan, Union of Comoros: Centre National de Documentation et de Recherche Scientifique (CNDRS) of Union des Comores: Export permit CNDRS 021/10.
5. Mahé, Seychelles Archipelago: Direction of Wildlife, Trade and Conservation Section and the Ministry of Environment and Energy of Republic of Seychelles: Export permits Agreement of 5 March 2014. CITES permit from the Republic of Seychelles was issued for export (permit N°1772) to CRVOI on La Réunion.
6. Mauritius: National Park and Conservation Service for authorization of Mauritius: Memorandum of agreement for the supply of biological material by Government of Mauritius, signed 17 December 2010 and 09 January 2013. CITES permit from the Mauritian national authority was issued for tissue export (permit MU120933) to CRVOI laboratory on La Réunion.
