# Supplementary material for: Serological Evidence of Lyssaviruses among Bats on Southwestern Indian Ocean Islands
Source: PLoS One. 2016 Aug 8;11(8):e0160553. doi: 10.1371/journal.pone.0160553 (PMC4976896; doi:10.1371/journal.pone.0160553)
Supplement: S2 Text — Ethical procedure applied for bat sampling on Madagascar, La Réunion, Mayotte, Anjouan, Mahé and Mauritius. (DOCX) [file pone.0160553.s010.docx]

**S2 Text**. Ethical clearance information

The procedures performed in this study were not subjected to the approval of an ethics committee or to specific national or international regulation at the time of sampling.

This study was conducted in strict accordance with the terms of research and permits issued by all national authorities.

Except for *Pteropus seychellensis* from Mayotte, which were released at the site of capture after blood and swab samples were collected, all bats used in this study were captured, manipulated, and dispatched (without sedation) by cardiac puncture following the guidelines accepted by the scientific community for the handling of wild mammals. In all cases, cardiac puncture resulted in the death of the animals and blood samples varied based on the size of the animal. For larger bodied bats, specifically members of the genus *Pteropus,* individuals were injected parenterally with euthanasia compound (sodium pentobarbital) before the cardiac puncture procedure.
